# New Samarium(III), Gadolinium(III), and Dysprosium(III) Complexes of Coumarin-3-Carboxylic Acid as Antiproliferative Agents

**DOI:** 10.1155/2007/15925

**Published:** 2007-03-01

**Authors:** Irena Kostova, Georgi Momekov, Peya Stancheva

**Affiliations:** ^1^Department of Chemistry, Faculty of Pharmacy, Medical University, 2 Dunav Street, 1000 Sofia, Bulgaria; ^2^Department of Pharmacology and Toxicology, Faculty of Pharmacy, Medical University, 2 Dunav Street, 1000 Sofia, Bulgaria; ^3^Department of Inorganic Chemistry, University of Sofia, 1000 Sofia, Bulgaria

## Abstract

New complexes of samarium(III), gadolinium(III), and dysprosium(III) with coumarin-3-carboxylic acid (HCCA) were prepared by the reaction of the ligand with respective metal nitrates in ethanol. The structures of the final complexes were determined by means of physicochemical data, elemental analysis, IR and Raman spectra. The metal-ligand binding mode in the new Ln(III) complexes of coumarin-3-carboxylic acid was elucidated. The vibrational study gave evidence for bidentate coordination of CCA^−^ to Ln(III) ions through the carbonylic oxygen and the carboxylic oxygen atoms. The complexes were tested for antiproliferative activitiy on the chronic myeloid leukemia-derived K-562, overexpressing the BCR-ABL fusion protein. Cytotoxicity towards tumor cells was determined for a broad concentration range. The samarium salt exerted a very weak antiproliferative effect on these cells. This is in contrast to the lanthanide complexes, especially samarium complex, which exhibited potent antiproliferative activity. The present study confirms our previous observations that the lanthanide complexes of coumarins exhibit antiproliferative activity towards K-562 cell line.

## 1. INTRODUCTION

Coumarin (1,2-benzopyrone) is structurally the least complex member of a large class of compounds known as benzopyrones [1]. The biological activities of coumarin and related compounds are multiple and include antithrombotic activity [2] and antimicrobial properties [3]. In addition, coumarins have been shown to inhibit N-methyl-N-nitrosourea, aflatoxin B1 and 7,12-dimethylbenz(a)anthracene-induced mammary carcinogenesis in rats [4, 5]. More recently, coumarin derivatives have been evaluated in the treatment of human immunodeficiency virus, due to their ability to inhibit human immunodeficiency virus integrase [6, 7]. Since the late 1980s, a number of in vitro and in vivo studies have investigated the possible use of coumarins in the treatment of cancer [8]. The in vitro effects of coumarins on the growth of renal cell carcinoma that derived cell lines showed that coumarin and 7-hydroxycoumarin were potent cytotoxic and cytostatic agents [9]. Several authors have reported on the use of coumarin (1,2-benzopyrone), or its metabolite 7-hydroxycoumarin, for the treatment of some human carcinomas [10–13]. There are several reports indicating that some coumarin compounds, including coumarin and 7-hydroxycoumarin, inhibit cell growth of cell lines of various types of cancer [14–18].

The coumarin derivatives have been the focus of our recent research concerning the design of new cytotoxic agents. It is well known that many investigations have proved that binding of a drug to a metalloelement enhances its activity and, in some cases, the complex possesses even more healing properties than the parent drug. This has prompted us to investigate the metal binding properties of several coumarin derivatives. We have recently reported the synthesis of lanthanide(III) complexes with some coumarins and the study of their anticancer activity [19–29]. In previous works [19–29], we investigated the coordination behavior of some 4-hydroxycoumarins with cerium(III), lanthanum(III), and neodymium(III). In the course of these studies, considering that lanthanides(III) have an interesting but not well-known biological role in living organisms as trace elements, we have investigated the coordination properties of a series of other lanthanides(III) with coumarin derivatives.

**Figure 1:**
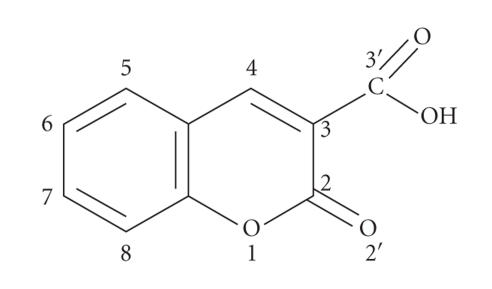
The structure of the ligand (HCCA, coumarin-3-carboxylic acid).

Thus, the aim of this work is to synthesize and characterize complexes of samarium(III), gadolinium(III), and dysprosium(III) with coumarin-3-carboxylic acid (see Figure 1) and to determine the antiproliferative effects of these complexes against the chronic myeloid leukemia-derived K-562. The cell line is characterized with a strong expression of BCR-ABL fusion protein (a constitutive nonreceptor tyrosine kinase) which determines the low responsiveness of these cells to proapoptotic stimuli [30].

## 2. METHODS

### 2.1. Chemistry

The compounds used for preparing the solutions were Merck products, p.a. grade: Sm(NO_3_)_3_ · 6H_2_O, Gd(NO_3_)_3_ · 6H_2_O, and Dy(NO_3_)_3_ · 5H_2_O. Coumarin-3-carboxylic acid (Figure 1) was used for the preparation of metal complexes as a ligand.

The complexes were synthesized by reaction of samarium(III), gadolinium(III), and dysprosium(III) salts and the ligand, in amounts equal to metal: ligand molar ratio of 1 : 2. The synthesis of the complexes was made in different ratios (1 : 1, 1 : 2, 1 : 3) but in all the cases the product was with the composition 1 : 2. The complexes were prepared by adding ethanol solutions of Ln(III) salts to ethanol solutions of the ligand. The reaction mixture was stirred with an electromagnetic stirrer at 25°C for one hour. At the moment of mixing of the solutions, precipitates were obtained. The precipitates were filtered, washed several times with water and ethanol, and dried in a desiccator to constant weight.

The complexes were insoluble in water, methanol, and ethanol and well soluble in DMSO.

The carbon, hydrogen, and nitrogen contents of the compounds were determined by elemental analysis. The water content was determined by Metrohn Herizall E55 Karl Fisher Titrator and was confirmed by TGA.

IR spectra (Nujol) were recorded on IR-spectrometer FTIR-8101M Shimadzu. The Raman spectra of the ligand and their new Ln(III) complexes were recorded with a Dilor microspectrometer (Horiba-Jobin-Yvon, model LabRam) equipped with a 1800 grooves/mm holographic grating. The 514.5 nm line of an argon ion laser (Spectra Physics, model 2016) was used for the probes excitation. The spectra were collected in a backscattering geometry with a confocal Raman microscope equipped with an Olympus LMPlanFL 50x objective and with a resolution of 2 cm^−1^. The detection of Raman signal was carried out with a Peltier-cooled CCD camera. The laser power of 100 mW was used in our measurements.

### 2.2. Pharmacology

The antiproliferative effects of the tested lanthanide complexes and of the corresponding nitrate salts were assessed on the chronic myeloid leukemia-derived K-562. The cells were maintained as suspension-type cultures in a controlled environment: RPMI 1640 medium (Sigma), with 10% heat inactivated fetal bovine serum (Sigma) and 2 mM L-glutamine (Sigma), in a “Heraeus” incubator with humidified atmosphere and 5% carbon dioxide, at 37°C. In order to keep the cells in log phase, cell suspension was discarded 2 or 3 times per week and the cell culture was refed with fresh RPMI-1640 aliquots.

The cell viability was determined using the MTT-dye reduction assay. Briefly, exponentially growing cells were seeded in 96-well microplates (100 *μ*l/well) at a density of 1 × 10^5^ cells per ml and after 24-hour incubation at 37°C they were exposed to various concentrations of the lanthanide complexes for 48 hours. For each concentration a set of 6 wells was treated. After the incubation with the test compounds MTT solution (10 mg/ml in PBS) was added (10 *μ*l/well). The plates were further incubated for 4 hours at 37°C and the formazan crystals formed were dissolved through addition of 100 *μ*l/well 5% solution of formic acid in 2-propanol (Merck). The absorption of the samples was then measured using an ELISA reader (Uniscan Titertec) at wavelength of 580 nm. The blank solution consisted of 100 *μ*l RPMI 1640 medium (Sigma), 10 *μ*l MTT stock, and 100 *μ*l 5% formic acid in 2-propanol. The survival fractions were calculated as percentage of the untreated control using the formula

(1) $$\text{SF}\;\%=\frac{{\text{A}}_{\text{test}}}{{\text{A}}_{\text{control}}}\times 100$$,

where A_test_ is the average value for the absorption at a given concentration and A_control_ is the average absorption of the untreated control, respectively.

The stock solutions of the tested lanthanide complexes (at 20 mM) were freshly prepared in DMSO, and thereafter consequently diluted in RPMI-1640 medium, in order to achieve the desired final concentrations. At the final dilutions obtained, the concentration of DMSO never exceeded 1%. The stock solutions (20 mM, in water) of the nitrate salts of the lanthanides were freshly prepared and following antibacterial filtration they were accordingly diluted in RPMI-1640 medium.

Data processing, generation of dose-response curves, and IC_50_ calculations were performed using Microsoft Excel and Microcal Origin software for PC.

## 3. RESULTS AND DISCUSSION

### 3.1. Chemistry

The new complexes were characterized by elemental analysis. The metal ions were determined after mineralization. The water content in the complexes was determined by Karl Fisher analysis. The nature of the complexes was confirmed by IR and Raman spectroscopy.

The data of the elemental analysis of the compounds obtained serving as a basis for the determination of their empirical formulas and the results of the Karl Fisher analysis are presented in Table 1. There is good agreement between the calculated and the found values.

The mode of bonding of the ligand to samarium(III), gadolinium(III), and dysprosium(III) ions was elucidated by recording the IR and Raman spectra of the complexes as compared with those of the free ligand. The vibrational spectra of the complexes showed new bands in comparison with these of the free ligand which have been assigned to the rocking, waggling, and metal-oxygen stretching vibrations.

### 3.2. Vibrational analysis

**Figure 2:**
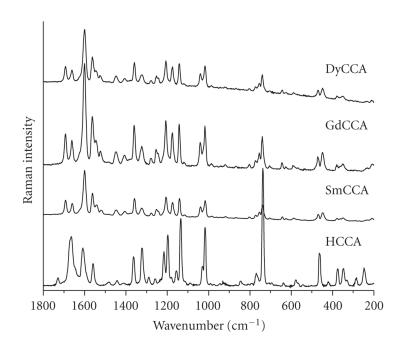
Experimental FT-Raman spectra of coumarin-3-carboxylic acid (HCCA) and its Sm(III), Gd(III), and Dy(III) complexes in the range 1800–200 cm^−1^.

Depending on the orientation of the two donor groups, C=O and COO^−^, different binding of the anion of coumarin-3-carboxylic acid (CCA^−^) is possible. However, in most of the known lanathanide complexes, the metal-ligand interaction is mainly electrostatic by nature. To help further the binding mode elucidation in the new Sm(III), Gd(III), and Dy(III) complexes of HCCA, detailed vibrational analysis was performed on the basis of comparison of experimental vibrational spectra of HCCA and its Sm(III), Gd(III), and Dy(III) complexes with those theoretically predicted by us earlier [31] as well as with literature data about related compounds. The data of the experimental FT-IR and FT-Raman spectra of HCCA and its Sm(III), Gd(III), and Dy(III) complexes are given in Table 2. The FT-Raman spectra of the ligand and its Ln(III) complexes are presented in Figure 2.

The broadband at 3176 cm^−1^ in the IR spectrum of the ligand was assigned to the *ν* (OH) vibrational mode. This band was not detected in the spectra of the complexes, indicating that the deprotonated ligand form participates in the complexes. The bands in the 3060–2920 cm^−1^ region were assigned to *ν* (CH) modes of HCCA. In the IR spectra of Sm(III), Gd(III), and Dy(III) complexes they remain almost unchanged.

The strong IR bands at 1746 cm^−1^ and 1685 cm^−1^ and the medium Raman bands at 1729, 1676, and 1663 cm^−1^ were assigned to *ν*(C=O) modes of the carboxylic and carbonylic groups, respectively. The high IR intensity of these bands retained in the spectra of Sm(III), Gd(III), and Dy(III) complexes, the *ν* (C=O)_carboxylic_ band was shifted to lower frequency (1703 cm^−1^, 1703 cm^−1^, 1705 cm^−1^ for Sm(III), Gd(III), and Dy(III) complexes, resp.), and the *ν* (C=O)_carbonylic_ band showed also a position change (1672 cm^−1^, 1672 cm^−1^, 1674 cm^−1^ for Sm(III), Gd(III), and Dy(III) complexes, resp.). The same shift effects were observed in the Raman spectra of the complexes.

In agreement with the literature data [31], the bands observed in the 1650–1330 cm^−1^ frequency range are due to the *ν* (CC) stretching vibrations of HCCA coumarin ring. The bands that are typical for the coumarin vibrations were not shifted significantly in the spectra of Sm(III), Gd(III), and Dy(III) complexes, which indicated that the Ln(III) cations did not produce substantial polarization on the coumarin ring. The strong IR (at 1613 and 1569 cm^−1^) and Raman (at 1608 and 1559 cm^−1^) bands are attributed to the *ν* (C=C) stretching vibrations of HCCA coumarin fragment. Their positions and intensities are almost retained and the second band is split in the complexes. The bands at 1489, 1453, and 1374 cm^−1^ (IR) and at 1483, 1442, and 1363 cm^−1^ (Raman), which also are assigned to the *ν* (CC) modes of HCCA, show shifts in the IR and Raman spectra of Sm(III), Gd(III), and Dy(III) complexes and at the same time the intensity of these bands increases. The induced polarization by Ln(III)−CCA interaction produces electron density distribution in the conjugated coumarin ring and as a result the *ν* (CC) frequencies change their positions and intensity.

The strong bands at 1228 cm^−1^ (IR spectrum of HCCA) and at 1216 cm^−1^ (Raman spectrum of HCCA) and the medium one at 989 cm^−1^, in the IR spectrum of HCCA, were assigned to the lactone *ν* (C−O) modes, respectively. In the complexes, these modes were shifted to lower frequency. In agreement with Ln(III)−O_carbonyl_ interaction, the induced polarization on CCA^−^ leads to changes of the C−O lactone bond lengths as well as of their frequencies in a direction mentioned above.

The following bands, observed in the IR spectra of the complexes, are assigned to the vibrational modes of the NO_3_ group: 1263 cm^−1^ (Sm complex), 1262 cm^−1^ (Gd complex), 1262 cm^−1^ (Dy complex) for *ν* (NO)_bonded_; 1053 cm^−1^ (Sm complex), 1049 cm^−1^ (Gd complex), 1049 cm^−1^ (Dy complex) for *δ* (ONO); 786 cm^−1^ (Sm complex), 791 cm^−1^ (Gd complex), 781 cm^−1^ (Dy complex) for *δ* (ONO); and 725 cm^−1^ (Sm complex), 725 cm^−1^ (Gd complex), 713 cm^−1^ (Dy complex) for *δ* (ONO). Some of them also appear in the Raman spectra of the complexes: 1040 cm^−1^ (Sm complex), 1040 cm^−1^ (Gd complex), 1043 cm^−1^ (Dy complex) for *δ* (ONO); 777 cm^−1^ (Sm complex), 772 cm^−1^ (Gd complex), 777 cm^−1^ (Dy complex) for *δ* (ONO). Because of the predominant electrostatic character of the Ln−O bonding, the bands corresponding to the *ν* (Ln−O) modes have low intensities, they are coupled with other modes and hence, their assignment is unreliable. The doublet bands observed in the IR spectra of the complexes at 767, 748 cm^−1^ (Sm complex), 768, 749 cm^−1^ (Gd complex), 766, 750 cm^−1^ (Dy complex), the bands at 459 cm^−1^ (Sm complex), 457 cm^−1^ (Gd complex), 457 cm^−1^ (Dy complex), and the bands at 449 cm^−1^ for Sm(III), Gd(III), and Dy(III) complexes were assigned to *ν* (Ln−O)_carboxylic_ and *ν* (Ln−O)_carbonylic_ modes, respectively.

On the basis of the above-detailed vibrational study we can conclude that the metal-ligand bonding in Ln(III) complexes of coumarin-3-carboxylic acid appeared to be strongly ionic with very small donor-acceptor character. The vibrational study gave evidence for bidentate coordination of CCA^−^ to Sm(III), Gd(III), and Dy(III) ions through the carbonylic oxygen and the carboxylic oxygen.

A survey of the ^1^H NMR spectral data reveals downfield chemical shifts of the protons in the Ln(III) complexes spectra relative to the free ligand. The resonances due to protons of the ligand are considerably broadened and shifted indicating complexation. The ligand shows a peak at 13.2 ppm due to the carboxylic proton [31]. This peak is absent in the spectra of the complexes due to the deprotonation of the carboxylic group. In the ^13^C NMR spectra of the complexes, the largest upfield chemical shifts are observed for the carbon atoms which are neighbors of the carboxylic and carbonylic oxygens and this finding confirms their participation in Ln(III)−CCA interaction.

The solvent DMSO was used for the NMR measurements because the solubility of the complexes in noncoordinating solvents was too low. DMSO is well known as a very reactive agent. DMSO molecule could indeed bind to the metal and give rise to equilibrium, fast on the NMR time scale, involving the DMSO molecule as an additional ligand, because of the high coordination number of Ln(III). Taking into consideration the obtained data, we can say the following. The results of NMR spectra, discussed above, and the results of the pharmacological activity, presented below, all made in DMSO, give us reason to suggest that in these conditions (in the solution of DMSO) the complexes are present. The stability of the complexes is of great interest with respect to their further pharmacological properties (not cytotoxicity test) and will be the subject of coming investigations which are in progress.

Our previous molecular electrostatic potential (MEP) study on the preferred reactive sites of CCA^−^ in the gas phase and in solution revealed two regions suitable for electrophilic attack and binding: between the deprotonated carboxylic and the carbonylic oxygens and between the carboxylic oxygens [31, 32]. To suggest the binding mode of HCCA, a detail theoretical and vibrational investigation based on Raman, FTIR, and DFT/B3LYP/SVP studies of HCCA, its deprotonated form (CCA^−^), KCCA, and Ln(CCA)_2_(NO_3_)(H_2_O) species, was performed. Two bidentate binding modes of CCA^−^ to Ln(III) were modeled: (1) through the deprotonated carboxylic and the carbonylic oxygens and (2) through both carboxylic oxygens. The vibrational analysis and the electronic energy calculations pointed to the first binding as more probable. On the basis of detailed DFT study of the vibrational behavior of HCCA, CCA^−^, KCCA, and Ln(CCA)_2_(NO_3_)(H_2_O) species and comparison of the theoretical and experimental vibrational spectra, it was established that CCA^−^ is bidentate bound to Ln(III) through the carboxylic and the carbonylic atoms. As seen from the vibrational spectra, the NO_3_ group is bidentate coordinated, the calculated and experimental NO_3_ modes for the complexes were found at very similar wavenumbers, and the assignment of the NO_3_ vibrational modes is in good agreement with literature data [31, 32].

Moreover, the metal-ligand binding mode of coumarin-3-carboxylic acid (HCCA) was recently explained by us through modeling of the Ln(III)-coumarin-3-carboxylic acid structures, where Ln = La, Ce, Nd [31, 32]. It was suggested that coumarin-3-carboxylic acid binds to the Ln(III) ions through both oxygen atoms of the carboxylic and carbonylic groups from the ligands and through the oxygen atoms of NO_3_^−^, and thus, the central ion Ln(III) is six-coordinated. The NBO analysis of the complexes suggests predominantly ionic character of the Ln−CCA bond with slight ligand-metal charge transfer [31, 32].

Nevertheless, we have to take into consideration that the coordination number 6 for these central metal ions indeed is too low, but not impossible for lanthanide(III) ions [19–29, 31, 32]. One plausible mode of coordination might involve also the water molecules, which is typical for coordination compounds of lanthanides.

On the bases of our experimental spectral data and our theoretical density function calculations [31, 32], we were able to suggest the most probable structure of these Ln(III) complexes.

### 3.3. Pharmacology

**Figure 3:**
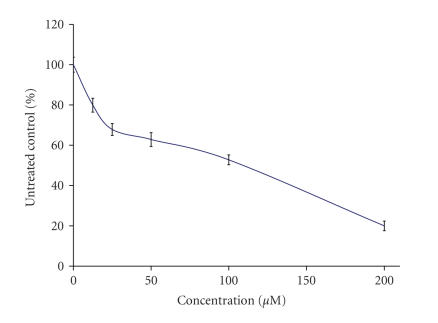
Cytotoxic effect of Sm(CCA)_2_(NO_3_) · H_2_O on the chronic myeloid leukemia-derived K-562 cell line after 48-hour exposure, as assessed by the MTT-dye reduction assay. Each data point represents the mean ± SD (*n* ≥ 6).

**Figure 4:**
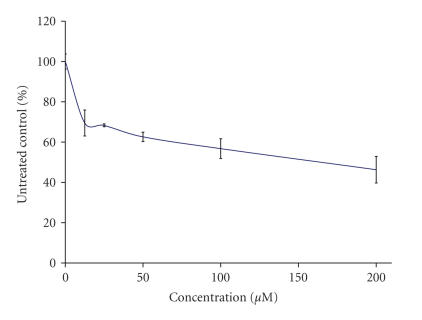
Cytotoxic effect of Gd(CCA)_2_(NO_3_) · H_2_O on the chronic myeloid leukemia-derived K-562 cell line after 48-hour exposure, as assessed by the MTT-dye reduction assay. Each data point represents the mean ± SD (*n* ≥ 6).

**Figure 5:**
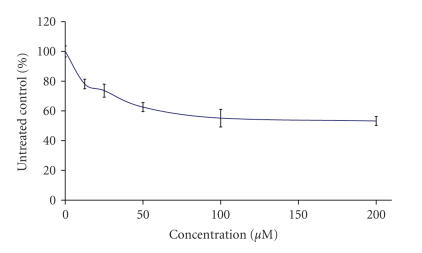
Cytotoxic effect of Dy(CCA)_2_(NO_3_) · H_2_O on the chronic myeloid leukemia-derived K-562 cell line after 48-hour exposure, as assessed by the MTT-dye reduction assay. Each data point represents the mean ± SD (*n* ≥ 6).

**Figure 6:**
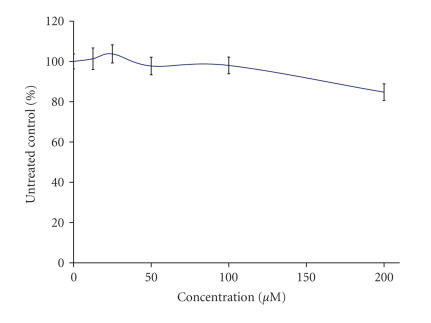
Cytotoxic effect of Sm(NO_3_)_3_ · 6H_2_O on the chronic myeloid leukemia-derived K-562 cell line after 48-hour exposure, as assessed by the MTT-dye reduction assay. Each data point represents the mean ± SD (*n* ≥ 6).

**Figure 7:**
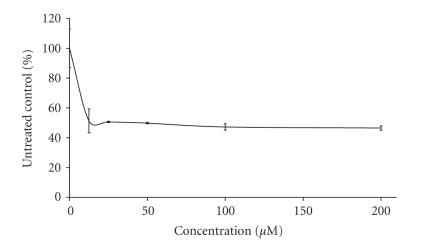
Cytotoxic effect of Gd(NO_3_)_3_ · 6H_2_O on the chronic myeloid leukemia-derived K-562 cell line after 48-hour exposure, as assessed by the MTT-dye reduction assay. Each data point represents the mean ± SD (*n* ≥ 6).

**Figure 8:**
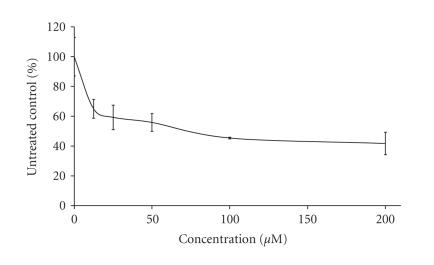
Cytotoxic effect of Dy(NO_3_)_3_ · 5H_2_O on the chronic myeloid leukemia-derived K-562 cell line after 48-hour exposure, as assessed by the MTT-dye reduction assay. Each data point represents the mean ± SD (*n* ≥ 6).

The preliminary pharmacological screening performed revealed that all of the lanthanide complexes exerted antiproliferative effects against the chronic myeloid leukemia-derived K-562 line in a concentration-dependent matter, which enabled the construction of concentration response curves as depicted on Figures 3, 4, 5, 6, 7, 8 and Table 3. In contrast to the Sm(III) and Gd(III) complexes with coumarin-3-carboxylic acid, Dy(CCA)_2_(NO_3_) · H_2_O failed to induce 50% inhibition of the cellular proliferation within the concentration range under investigation.

Interestingly, the Gd(III) and Dy(III) nitrates were found to exert considerable antiprolferative effects (Figures 6, 7, 8), superior to those of the complexes thereof. In contrast, despite the considerable activity of the samarium complex, the corresponding nitrate salt Sm(NO_3_)_3_ · 6H_2_O (Figure 6) caused only marginal inhibitory effects against K-562 (Figure 3).

## 4. CONCLUSION

The results from this study demonstrate the antiprolferative potential of three novel lanthanide coordination compounds of coumarin-3-carboxylic acid derivatives, in line with our preceding papers concerning the activity of lanthanide (Ce(III), La(III), and Nd(III)) coordination compounds with diverse coumarin ligands [19–29]. In our hands, the samarium(III) complex of coumarin-3-carboxylic acid proved to be the most active antiproliferative agent among the novel complexes and thus it necessitates further more detailed pharmacological evaluation. The complex formation proved to be detrimental for the efficacy of Gd(III) and Dy(III) compounds as in both cases the nitrates exerted superior efficacy versus the corresponding coordination compounds.

## Figures and Tables

**Table 1:** Elemental analysis of Ln(III) complexes of coumarin-3-carboxilic acid. HCCA = C_10_H_6_O_4_; CCA = C_10_H_5_O_4_^−^.

Compound	Calculated/found (%)				

Formulae	C	H	N	H_2_O	Ln

Sm(CCA)_2_(NO_3_) · H_2_O	39,47/	1,97/	2,30/	2,96/	24,67/
	39,09	2,02	2,77	2,73	24,25

Gd(CCA)_2_(NO_3_) · H_2_O	39,02/	1,95/	2,28/	2,92/	25,53/
	39,38	2,28	2,49	2,69	25,22

Dy(CCA)_2_(NO_3_) · H_2_O	38,71/	1,94/	2,26/	2,90/	26,13/
	38,90	1,86	2,05	2,58	25,84

**Table 2:** Experimental vibrational frequencies of HCCA and its Ln(III) complexes.

HCCA		Sm-HCCA		Gd-HCCA		Dy-HCCA		Assignments
IR	Raman	IR	Raman	IR	Raman	IR	Raman	

3176 w	—	—	—	—	—	—	—	*ν* (OH)_coum_
3057 w	3066 w	3050 w	—	3051 w	—	3054 w	—	*ν* (CH)
2956 w	—	2953 vw	—	2953 vw	—	2953 w	—	*ν* (CH)
2926 w	—	2924 w	—	2922 w	—	2924 w	—	*ν* (CH)
1746 vs	1729 m	1703 m	1692 m	1703 s	1692 m	1705 vs	1690 m	*ν* (C=O)_carboxylic_
1685 s	1676 m	1672 vs	1660 m	1672 s	1659 m	1684 sh	1659 m	*ν* (C=O)_carbonylic_
—	1663 m	—	—	—	—	1674 s	—	—
1613 s	1608 vs	1613 vs	1600 vs	1613 vs	1600 vs	1615 vs	1600 vs	*ν* (CC)
1569 s	1559 m	1572 s	1561 s	1572 s	1561 s	1581 vs	1563 s	*ν* (CC)
—	—	1553 s	1540 m	1556 s	1540 m	1556 s	1538 m	—
1489 w	1483 w	1510 m	1445 m	1512 m	1448 m	1485 vw	1447 m	*ν* (CC) + *δ* (CCH)_ip_
1453 w	1442 w	1456 m	1406 w	1458 m	1406 m	1458 m	1405 w	*ν* (CC) + *δ* (CCH)_ip_
—	—	1408 s	—	1410 s	1380 br	1408 s	—	—
1422 s	1413 vw	—	—	—	—	—	—	*δ* (COH)_ip_
1374 m	1363 s	1355 w	1323 m	1356 w	1323 m	1385 vs	1320 m	*ν* (CC) + *δ* (CCH)_ip_
1228 s	1216 s	—	1204 s	—	1207 s	1215 w	1204 s	*ν*(C−O)_lactone_
1208 s	1197 vs	1299 sh	1275 w	1287 sh	1278 w	1255 m	1278 w	*ν*(C−O)_carboxylic_
—	—	1282 s	—	1283 m	—	—	—	—
—	—	1263 m	—	1262 m	—	1262 m	—	*ν* (NO)^as^
—	—	1053 w	1040 m	1049 w	1040 m	1049 w	1043 m	*δ* (ONO)
989 m	—	976 w	984 w	971 vw	—	964 w	986 w	*δ* (CCH)_op_
802 s	—	—	—	—	—	—	—	*δ* (COH)_op_
—	—	786 w	777 w	791 vw	772 w	781 w	777 w	*δ* (ONO)
—	—	767 m	—	768 m	—	766 s	—	*δ* (OCO)_ip(cabox)_
—	—	748 w	740 m	749 w	740 m	750 w	740 m	+*ν* (Ln−O)_carboxylic_
—	—	725 vw	—	725 vw	—	713 vw	—	*δ* (ONO)
—	—	459 w	468 w	457 w	475 w	457 w	469 w	*ν* (Ln−O)_carbonylic_
—	—	449 w	—	449 w	—	449 w	—	—
—	—	No data	209 w	No data	206 w	No data	211 vw	*ν* (Ln−O_NO3_)

**Table 3:** Relative potency of the investigated compounds in the panel of human tumor cell line K-562, following 48-hour treatment (MTT-dye reduction assay).

Compound	IC_50_ value^(a)^

Sm(CCA)_2_(NO_3_) · H_2_O	108.39 ± 6.9 *μ*M
Gd(CCA)_2_(NO_3_) · H_2_O	164.52 ± 11.23 *μ*M
Dy(CCA)_2_(NO_3_) · H_2_O	>200 *μ*M
Sm(NO_3_)_3_ · 6H_2_O	>200 *μ*M
Gd(NO_3_)_3_ · 6H_2_O	41.35 ± 5.9 *μ*M
Dy(NO_3_)_3_ · 5H_2_O	76.78 ± 4.72 *μ*M

^(a)^Data represent the arithmetic mean ± standard deviation of six independent experiments.

## References

[1] Egan D, O'Kennedy R, Moran E, Cox D, Prosser E, Thornes RD (1990). The pharmacology, metabolism, analysis, and applications of coumarin and coumarin-related compounds. *Drug Metabolism Reviews*.

[2] Hoult JRS, Payá M (1996). Pharmacological and biochemical actions of simple coumarins: natural products with therapeutic potential. *General Pharmacology*.

[3] Laurin P, Ferroud D, Klich M (1999). Synthesis and in vitro evaluation of novel highly potent coumarin inhibitors of gyrase B. *Bioorganic and Medicinal Chemistry Letters*.

[4] Matsumoto A, Hanawalt PC (2000). Histone H3 and heat shock protein GRP78 are selectively cross-linked to DNA by photoactivated gilvocarcin V in human fibroblasts. *Cancer Research*.

[5] Kelly VP, Ellis EM, Manson MM (2000). Chemoprevention of aflatoxin B_1_ hepatocarcinogenesis by coumarin, a natural benzopyrone that is a potent inducer of aflatoxin B_1_-aldehyde reductase, the glutathione *S*-transferase A5 and P1 subunits, and NAD(P)H: quinone oxidoreductase in rat liver. *Cancer Research*.

[6] Kirkiacharian S, Thuy DT, Sicsic S, Bakhchinian R, Kurkjian R, Tonnaire T (2002). Structure-activity relationships of some 3-substituted-4-hydroxycoumarins as HIV-1 protease inhibitors. *Il Farmaco*.

[7] Yu D, Suzuki M, Xie L, Morris-Natschke SL, Lee K-H (2003). Recent progress in the development of coumarin derivatives as potent anti-HIV agents. *Medicinal Research Reviews*.

[8] Marshall EM, Ryles M, Butler K, Weiss L (1994). Treatment of advanced renal cell carcinoma (RCC) with coumarin and cimetidine: longterm follow-up of patients treated on a phase I trial. *Journal of Cancer Research and Clinical Oncology*.

[9] Conley D, Marshall EM (1987). Effects of coumarin on human tumour cell growth and cell cycle analysis in vitro. *Proceedings of the American Association for Cancer Research*.

[10] Thornes RD, Daly L, Lynch G (1994). Treatment with coumarin to prevent or delay recurrence of malignant melanoma. *Journal of Cancer Research and Clinical Oncology*.

[11] Marshall ME, Mohler JL, Edmonds K (1994). An updated review of the clinical development of coumarin (1,2-benzopyrone) and 7-hydroxycoumarin. *Journal of Cancer Research and Clinical Oncology*.

[12] Mohler JL, Williams BT, Thompson IM, Marshall ME (1994). Coumarin (1,2-benzopyrone) for the treatment of prostatic carcinoma. *Journal of Cancer Research and Clinical Oncology*.

[13] von Angerer E, Kager M, Maucher A (1994). Antitumour activity of coumarin in prostate and mammary cancer models. *Journal of Cancer Research and Clinical Oncology*.

[14] Lake BG (1999). Coumarin metabolism, toxicity and carcinogenicity: relevance for human risk assessment. *Food and Chemical Toxicology*.

[15] Marshall ME, Butler K, Fried A (1991). Phase I evaluation of coumarin (1,2-benzopyrone) and cimetidine in patients with advanced malignancies. *Molecular Biotherapy*.

[16] Marshall ME, Kervin K, Benefield C (1994). Growth-inhibitory effects of coumarin (1,2-benzopyrone) and 7-hydroxycoumarin on human malignant cell lines in vitro. *Journal of Cancer Research and Clinical Oncology*.

[17] Myers RB, Parker M, Grizzle WE (1994). The effects of coumarin and suramin on the growth of malignant renal and prostatic cell lines. *Journal of Cancer Research and Clinical Oncology*.

[18] Kawaii S, Tomono Y, Ogawa K, Sugiura M, Yano M, Yoshizawa Y (2001). The antiproliferative effect of coumarins on several cancer cell lines. *Anticancer Research*.

[19] Kostova I, Manolov I, Nicolova I, Konstantinov S, Karaivanova M (2001). New lanthanide complexes of 4-methyl-7-hydroxycoumarin and their pharmacological activity. *European Journal of Medicinal Chemistry*.

[20] Kostova I, Manolov I, Konstantinov S, Karaivanova M (1999). Synthesis, physicochemical characterisation and cytotoxic screening of new complexes of cerium, lanthanum and neodymium with Warfarin and Coumachlor sodium salts. *European Journal of Medicinal Chemistry*.

[21] Manolov I, Kostova I, Konstantinov S, Karaivanova M (1999). Synthesis, physicochemical characterization and cytotoxic screening of new complexes of cerium, lanthanum and neodymium with Niffcoumar sodium salt. *European Journal of Medicinal Chemistry*.

[22] Manolov I, Kostova I, Netzeva T, Konstantinov S, Karaivanova M (2000). Cytotoxic activity of cerium complexes with coumarin derivatives. Molecular modeling of the ligands. *Archiv der Pharmazie: Pharmaceutical and Medicinal Chemistry*.

[23] Kostova I, Manolov I, Nicolova I, Danchev ND (2001). New metal complexes of 4-methyl-7-hydroxycoumarin sodium salt and their pharmacological activity. *Il Farmaco*.

[24] Kostova I, Manolov I, Karaivanova M (2001). Synthesis, physicochemical characterization, and cytotoxic screening of new zirconium complexes with coumarin derivatives. *Archiv der Pharmazie: Pharmaceutical and Medicinal Chemistry*.

[25] Kostova I, Manolov I, Momekov G (2004). Cytotoxic activity of new neodymium (III) complexes of bis-coumarins. *European Journal of Medicinal Chemistry*.

[26] Kostova I, Trendafilova N, Momekov G (2005). Theoretical and spectroscopic evidence for coordination ability of 3,3′-benzylidenedi-4-hydroxycoumarin. New neodymium (III) complex and its cytotoxic effect. *Journal of Inorganic Biochemistry*.

[27] Kostova I, Momekov G, Zaharieva M, Karaivanova M (2005). Cytotoxic activity of new lanthanum (III) complexes of bis-coumarins. *European Journal of Medicinal Chemistry*.

[28] Kostova I, Trendafilova N, Mihaylov T (2005). Theoretical and spectroscopic studies of pyridyl substituted bis-coumarins and their new neodymium (III) complexes. *Chemical Physics*.

[29] Kostova I, Manolov I, Momekov G, Tzanova T, Konstantinov S, Karaivanova M (2005). Cytotoxic activity of new cerium (III) complexes of bis-coumarins. *European Journal of Medicinal Chemistry*.

[30] Drexler HG, MacLeod RAF, Uphoff CC (1999). Leukemia cell lines: in vitro models for the study of Philadelphia chromosome-positive leukemia. *Leukemia Research*.

[31] Mihaylov Tz, Trendafilova N, Kostova I, Georgieva I, Bauer G (2006). DFT modeling and spectroscopic study of metal-ligand bonding in La(III) complex of coumarin-3-carboxylic acid. *Chemical Physics*.

[32] Georgieva I, Trendafilova N, Kiefer W, Rastogi VK, Kostova I Vibrational and theoretical study of coumarin-3-carboxylic acid
binding mode in Ce(III) and Nd(III) complexes.

